# Symbiotic Navigation in Multi-Robot Systems with Remote Obstacle Knowledge Sharing

**DOI:** 10.3390/s17071581

**Published:** 2017-07-05

**Authors:** Abhijeet Ravankar, Ankit A. Ravankar, Yukinori Kobayashi, Takanori Emaru

**Affiliations:** Faculty of Engineering, Lab of Robotics and Dynamics, Hokkaido University, Sapporo 060-8628, Japan; ankit@eng.hokudai.ac.jp (A.A.R.); kobay@eng.hokudai.ac.jp (Y.K.); emaru@eng.hokudai.ac.jp (T.E.)

**Keywords:** robot path planning, multi-robot knowledge sharing, robots in sensor network

## Abstract

Large scale operational areas often require multiple service robots for coverage and task parallelism. In such scenarios, each robot keeps its individual map of the environment and serves specific areas of the map at different times. We propose a knowledge sharing mechanism for multiple robots in which one robot can inform other robots about the changes in map, like path blockage, or new static obstacles, encountered at specific areas of the map. This symbiotic information sharing allows the robots to update remote areas of the map without having to explicitly navigate those areas, and plan efficient paths. A node representation of paths is presented for seamless sharing of blocked path information. The transience of obstacles is modeled to track obstacles which might have been removed. A lazy information update scheme is presented in which only relevant information affecting the current task is updated for efficiency. The advantages of the proposed method for path planning are discussed against traditional method with experimental results in both simulation and real environments.

## 1. Introduction

In recent years, there has been a surge of autonomous service robots used for cleaning, surveillance, entertainment, and object delivery at hospitals, warehouses, and public places. Generally, multiple mobile robots are used as they have several advantages. Multiple robots can cover larger areas and execute work in parallel. Failure of one robot does not bring down the entire system. These mobile robots need to navigate from one place to another to provide services like cleaning, or object delivery. To do this, they are equipped with exteroceptive sensors like laser range finders and cameras to perceive the external world. The robots have software modules to process the data collected from these sensors for path planning, localization, mapping, and obstacle avoidance [1,2,3]. The environments in which these robots operate are often dynamic. For example, although there are fixed elements in the map like walls, the positions of other objects like furniture are not absolute and may change in the map with time. Some paths may be blocked temporarily due to cleaning or repair operations.

There is a perception limit to each robot due to the limitation of the sensors, and the robots are only aware of the changes in their own local vicinity. Hence, if a path at a remote location in the map is blocked, robots may not know it until they explicitly visit that location. Traditionally, if one robot has discovered that a path is blocked, this knowledge is only local to itself and it can plan a new path to the goal location. However, other robots do not have this information, and they plan their paths without considering the new obstacle. In a large environment in which multiple robots navigate long distances, this limitation severely affects the system’s performance, as more and more robots spend time in re-planning and navigating a new path from the blocked location to the goal.

**Figure 1:**
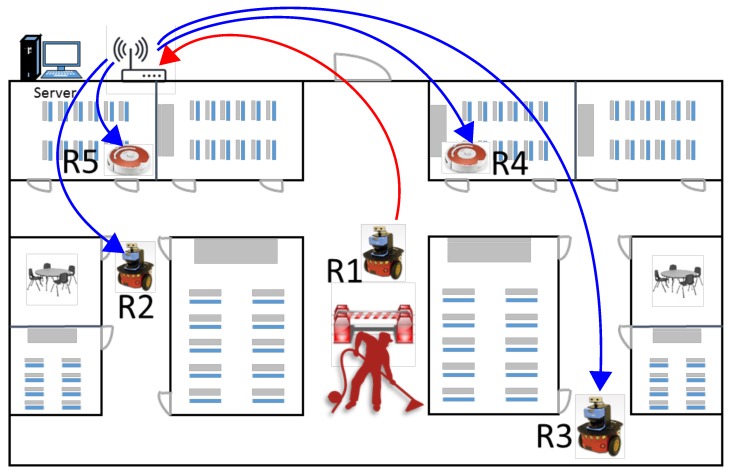
Symbiotic navigation in the sensor network. Robot R1 finds a path blocked and shares this information with other robots which can plan efficient paths considering the timely information.

This paper proposes a knowledge sharing system to solve the aforementioned problem, in which, if a robot finds a new obstacle in one of the paths of the map, it notifies other robots about the obstacle enabling them to consider the new information while planning their paths. Such notification is possible in a sensor network which enables robots to communicate with each other. This idea is graphically shown in Figure 1, in which robot R1 finds one of the paths blocked and communicates this information to other robots on the same network. The other robots thus have a timely information to consider while planning their paths. This is thus, a symbiotic navigation scheme in which robots help each other by providing timely information of the newly encountered obstacles to plan optimal paths. However, sharing the new obstacle information is difficult as each robot maintains its own map and there is a problem of correspondence of features. Moreover, different robots may maintain different types of maps, and the transience of the new obstacle in the map needs to be modeled. This work provides solutions for these problems, and discusses how the proposed method increases the efficiency of multi-robot navigation.

### State of the Art

Robot path planning is a well studied problem. The most widely used algorithms are A* algorithm [4], D* algorithm [5,6], probabilistic roadmap planner (PRM) [7], rapidly exploring random tree (RRT) [8,9], and potential fields [10] algorithms. A detailed summary of these algorithms can be found in [11,12,13]. Multi-robot planning is either centralized or decentralized. In centralized approaches [14], all the paths of all robots are calculated simultaneously. However, in decentralized approaches [15] each robot calculates its path individually. Multi-robot navigation in warehouse has been discussed in [16]. Avoiding multi-robot path conflicts has been discussed in [17]. Work in [18] proposes a decentralized belief system for collaboration between multiple robots in which different sources of uncertainty are considered to take robot action. A review of multi-robot navigation strategies can be found in [19,20].

In our previous work [21], we discussed the problem of multi-robot navigation in a camera sensor network, in which, robots communicate with the existing surveillance camera nodes fixed on the ceiling to detect far off obstacles and people moving in the back side of the robot. The work relied on vision sensors and the shared information was limited to that in the field-of-view of the cameras. The current work presents a new idea in which the robots themselves share information about the obstacles encountered in remote areas of the map. Information sharing between robots has been discussed in [22] in which two robots with different capabilities share information to match affordances, i.e., whether an object is graspable or moveable. Work in [23] proposes an algorithm which shares corresponding matches of an object over time by two robots to calculate an accurate relative localization. Visual information sharing has also been proposed in [24]. In [25], multiple robots share information and negotiate with each other to obtain a task and decide the executing sequence of sub-tasks. A protocol for sharing the region of interest between robots which observe different regions of the same scene to cooperate tasks efficiently has been proposed in [26,27]. Robo-soccer [28,29] is another area which heavily relies on information sharing between robots for success. Information sharing for pheromone based trailing of master-slave robots has also been proposed [30]. Regarding path planning in multi-robot scenarios, work in [31] presents an algorithm to efficiently avoid collision and collaboratively find an optimal path. The proposed work focuses on multiple robots sharing information about the dynamic changes in the remote area of the environment, enabling the robots to use updated and timely information to efficiently plan their paths.

The occurrence of obstacles in the map can be thought of as ‘events’, and many researchers have addressed event driven task scheduling in previous works. Event based systems are a type of reactive systems and have been widely used in control, digital signal processing, and communication systems. In distributed and wireless networks, they are characterized by efficient utilization of communication bandwidth, computation capability, and energy [32]. Work in [33] discusses a distributed and decentralized system with independent local observations. It was the first adoption of the concept of the level-crossing sampling to the selection of the hypotheses in sequential likelihood ratio test in the context of distributed decision systems. Work in [34] proposes the use of time dimension for information fusion for detection (binary hypothesis testing). In [35], a solution for event based control of mobile robots is presented for wireless environments. In a related work [36], a dynamic selection of appropriate threshold for send-on-delta [37] sampling is proposed. A send-on-delta [37] is an information collection strategy where sampling is triggered when specific events occur in the environment. A sensor fusion technique is presented in [38] to improve the localization of mobile robots. An experimental platform to communicate between multiple mobile robots in a wireless sensor network has been presented in [39]. It presents an event triggered communication and distributed control algorithm. Details of event based control techniques can be found in [32,40].

The rest of the paper is organized as follows. Section 2 deals with obstacle information on node representation of the path. Section 2.2 explains path planning on node map. Section 3 explains the obstacle removal and update in the map with lazy update scheme. Results are shown in Section 4, with simulation results shown in Section 4.1, and results in real environment shown in Section 4.2. Results are discussed in Section 5 along with the limitations of the proposed method. Finally, Section 6 concludes the paper.

## 2. Node Representation with Obstacle Information and Path Planning

This section describes the node representation of the map. We first explain the node map and how obstacles are represented in it. Later, we describe how path planning is done on the node map.

### 2.1. Node Map and Obstacle Information

This work assumes that the robots of the multi-robot system are on the same network and can communicate with each other and to a central server. Each robot is also assigned a unique robot-id ($R_{\mathrm{id}}$).

Each robot of a multi-robot system has a SLAM (Simultaneous Localization and Mapping) [41] unit which builds a map and localizes itself in it. The robots also update the positions of new entities in the map. The new entities could be the temporary obstacles, or new permanent features and both needs to be updated in the map for correct path planning. A robot estimates the absolute position ($x_{obs},y_{obs}$) of an obstacle in its map along with the uncertainty ($\Sigma_{obs}$) associated with it. This information about the new obstacle ($x_{obs},y_{obs},\Sigma_{obs}$) can directly be shared with other robots, however, there are certain disadvantages in doing so:

1. **Correspondence Problem**: Different robots may have built their maps from different starting locations. Therefore, a particular location $x^{1},y^{1}$ localized by one robot (say $R_{\mathrm{id}}=1$) might correspond to a different location $x^{2},y^{2}$ for another robot (say $R_{\mathrm{id}}=2$). Although there are techniques [42] to find the necessary translation and rotation required to transform one robot’s localized coordinates to another robot’s map coordinates, it takes time which can cause service delays.
2. **Diversity of Robot Specifications**: Different robots may have different types of sensors, software modules, and computation units. One robot may use a grid map while another robot may use a feature based map. Even for same type of sensors, their specifications (like accuracy, range) may vary. These differences make it difficult for the robots to utilize the directly transferred obstacle coordinates in a meaningful way.

**Figure 2:**
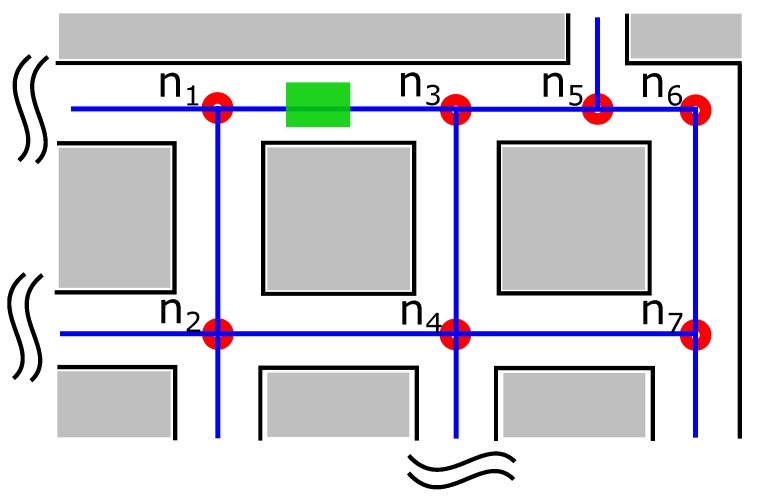
Node representation of path. The green block is the obstacle between nodes $n_{1}$ and $n_{3}$.

To overcome the aforementioned problems in sharing the obstacle information meaningfully, we use a node representation of the path. We define a node as a point of turn in a path of the map. All the paths of the map are represented as a network of these nodes. Figure 2 shows the node representation of the path. The nodes $n_{1},n_{2},\cdots ,n_{7}$ are the points of turns in the map. The line joining the two nodes is an edge. An edge $E_{\mathrm{ab}}$ is traversable if there is no obstacle between the nodes a and b, and not traversable if there is an obstacle. In Figure 2, the green block between nodes $n_{1}$ and $n_{3}$ represents and obstacle. Therefore, edge $E_{13}$ is not traversable, while others (ex: $E_{24}$) are.

A dictionary of edge information (E) is maintained for the entire map indicating which edges are traversable and which are not, in the form of key-value pairs. The keys are the edges of the map, and a value indicates whether the edge is traversable (0) or not (1). In case the path is blocked but partially traversable, a value of (−1) is used. For the path representation of Figure 2, the following dictionary is realized:

(1) $$E=\{\;\left\{E_{12}:0\right\},\left\{E_{24}:0\right\},\left\{E_{13}:1\right\},\cdots \;\}$$

For all the keys (edges) in E whose value is 1, i.e., edges who are blocked by new obstacles, following information about the obstacle is maintained in a tuple:

(2) $$D_{\mathrm{ab}}=\left(x_{i},y_{i},\Sigma_{i},d_{i},t_{i}\right),\;\;\;\;\;\;\;i\in \left\{1,2,\cdots ,n\right\}$$

where, for an *i*th obstacle, $x_{i}$, $y_{i}$ are the estimated coordinates of the obstacles with uncertainty $\Sigma_{i}$, $d_{i}$ is an array of the obstacle dimensions (length×width), and $t_{i}$ is the time-stamp at which the obstacle was last discovered.

With this representation, it becomes easier for a robot to share information with other robots. Even if the local maps maintained by the two robots differ by some rotation, translation, or scale, the nodes on the paths remains the same and information that there is an obstacle on one of the edges remains the same in both the local maps. The details of the obstacles are maintained by Equation (2).

Another benefit of this representation is that there is no need to maintain a global map. Instead, only the dictionary E shown in Equation (1) needs to be maintained. Although E may have a large number of nodes, only those edges whose value is 1, i.e., the edges over which there is an obstacle needs to be communicated to the robots. This saves a lot of communication bandwidth and ensures fast communication with the robots.

### 2.2. Path Planning on Node Map

If a robot ($R_{\mathrm{id}}$) finds a new obstacle across an edge $E_{\mathrm{ab}}$ such that the path is blocked, it updates its local dictionary E by setting $\left\{E_{\mathrm{ab}}:1\right\}$. This information is then send to the central server which updates its dictionary and broadcasts the update message to all the other robots ($R_{i},i\in \left\{1,2,\cdots ,n\right\},i\neq id$). Hence, irrespective of the current positions of the robots in the map, all the robots are informed that there is an obstacle on $E_{\mathrm{ab}}$ which is no longer traversable. As a concrete example, consider Figure 2. If a robot discovers that $E_{13}$ is blocked, it can share this information with all the robots via a central server.

With this updated information at disposal, other robots too can plan or change their path. For example, in case of Figure 2, if a robot’s initial path was $n_{2}\to n_{1}\to n_{3}$, it can change its path to $n_{2}\to n_{4}\to n_{3}$ to reach node $n_{3}$. In traditional robot navigation without knowledge sharing, the robot would have traversed node $n_{2}\to n_{1}$ and then discover that the path is blocked. It would then have to re-plan a new path to $n_{3}$. With the proposed knowledge sharing, the robots can know about the new and remote obstacles on the paths locally and without having to visit the remote locations. This improves the system performance.

For any path planning algorithm, it can be checked if the path is blocked. For example, A* [4] is a famous and commonly used algorithm for path planning. Let G=(V,E) is a graph with non-negative edge distances, and h is an admissible heuristic. If $S_{\mathrm{id}}$ is the start location and $G_{\mathrm{id}}$ be the goal location of a robot, d(v) is calculated as the shortest distance from $S_{\mathrm{id}}$ to v, and then d(v)+h(v) gives an estimate of the distance from $S_{\mathrm{id}}$ to v, and similarly from v to $G_{\mathrm{id}}$. The queue of nodes $Q=(V_{1},V_{2},\cdots ,V_{n})$ sorted by d(v)+h(v) is the A* path from $S_{\mathrm{id}}$ to $G_{\mathrm{id}}$. Once the path has been calculated, a check is performed to see if the path contains any nodes ($V_{i},i\in \left\{1,2,\cdots ,n\right\}$) which lie on the blocked edge $E_{\mathrm{blocked}}$. If so, then the route to the goal is re-planned considering the new obstacle. Even if the robot is currently in navigation, for any obstacle update, it can quickly be checked if the path is blocked or not, and navigation can be continued or a new path can be planned accordingly.

## 3. Obstacle Removal and Update

Most of the obstacles in the passages are temporary, and are removed after some time. Whether an obstacle is actually removed or not can only be known if a robot actually updates its map and informs other robots by setting the edge profile of that particular node (say $E_{\mathrm{ab}}$) to zero ($\left\{E_{\mathrm{ab}}:0\right\}$). However, there is no upper time bound of when a robot would actually visit the particular location and update its map. The obstacle might already have been removed by that time. This problem needs to be modeled mathematically.

A timestamp (*t*${}_{i}$) is maintained for an *i*th obstacle representing the time at which the obstacle was last seen. If an obstacle has recently been added to the map and a short time has elapsed since its addition, then the probability that it has not been removed is high. On the contrary, if a lot of time has elapsed since the addition of the obstacle, the probability that it still exists in the map is less. We model a confidence (*c*) measure which represents this probability $0\leq c_{th}\leq 1$. The maximum value of confidence is 1 and its value decreases with time. The robots assumes that the obstacle still exists in the map until the corresponding confidence has not dropped to below a threshold confidence ($C_{th}$). Depending on the nature of the environment, a threshold time ($t_{th}$) is chosen in which the confidence decays to $c_{th}$ value, and the time in which the confidence decays to zero is ($t_{z}$). To model the confidence decay, the following family of curves are chosen.

(3) $$c=1-{\left(\frac{t_{th}}{t_{z}}\right)}^{n}$$

The curves given by Equation (3) have the desired characteristic that for higher values of *n*, the curve flattens out more and delays confidence decay until the threshold time ($t_{th}$), and after that it decays quickly to zero in $t_{z}$ time. For a given $c_{th}$, $t_{th}$, and $t_{z}$, the value of the degree of the curve (*n*) can be found by solving Equation (3) as,

(4) $$\begin{array}{c} {\left(\frac{t_{th}}{t_{z}}\right)}^{n}=1-c_{th},\;\;\;\;\left(0\leq c_{th}\leq 1\right),\\ n\;\log\left(\frac{t_{th}}{t_{z}}\right)=\log\left(1-c_{th}\right),\\ \Rightarrow n=\frac{\log(1-c_{th})}{\log\left(\frac{t_{th}}{t_{z}}\right)}. \end{array}$$

**Figure 3:**
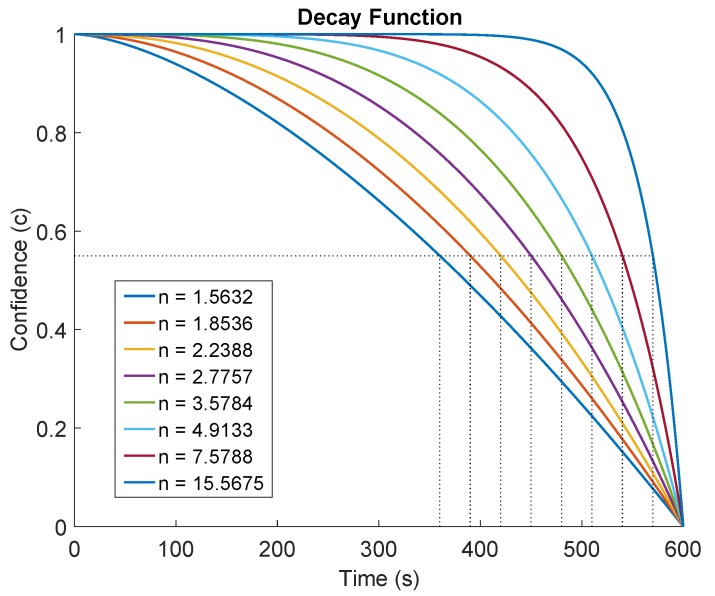
Confidence of temporary obstacle’s existence. The curve flattens out for higher values of *n* in Equation (3), taking more time to reach the threshold time.

Figure 3 shows the curves for the decay function given by Equation (3) for various values of *n*. The various curves have been generated for $c_{th}=0.65$ and $t_{z}=600$ s, for varying values of $t_{th}$ between 360 s to 570 s. It can be seen that the corresponding values of *n* can be found for different $t_{th}$ according to Equation (4). Moreover, as the value of *n* increases, the decay curves flatten out more taking more time to reach the threshold time, and then quickly decrease to zero.

For a given instantaneous value of confidence *c*, the elapsed time *t* is calculated from Equation (3) as,

(5) $$\begin{array}{c} t=e^{\frac{1}{n}\log\left(1-c\right)+\log\left(t_{z}\right)}. \end{array}$$

The time remaining ($t_{rem}$) to reach the threshold time ($t_{th}$) is,

(6) $$t_{rem}=t_{th}-e^{\frac{1}{n}\log\left(1-c\right)+\log\left(t_{z}\right)}.$$

In order to ensure a smooth robot operation in a sensor network in which multiple robots frequently inform each other about the new obstacle information, a ‘lazy update’ mechanism is proposed. If a robot receives an obstacle information update from another robot while it is navigating towards its goal location, then it would have to stop and update its map information which consumes time and computation. However, a ‘lazy update’ of obstacles is done, in which, a check is performed to see if the information received affects the current navigation towards the goal. In other words, update is performed ‘immediately’ only if the new obstacle information ($V_{b}$) relates to one of the nodes on the current path. If the queue of nodes on current navigation path are: $Q=(V_{1},V_{2},\cdots ,V_{n})$, map update is delayed if:

- The blocked node $V_{b}\notin Q$. In other words, the current path is not blocked.
- $V_{b}\in Q^{\prime }$ where the set of $Q^{\prime }$ nodes have already been traversed by the robot.

The new obstacle information is stored in a queue and updated when the robot has reached its goal. This ‘lazy update’ ensures smooth navigation and robots only update relevant information.

## 4. Results

### 4.1. Results in Simulation Environment

**Figure 4:**
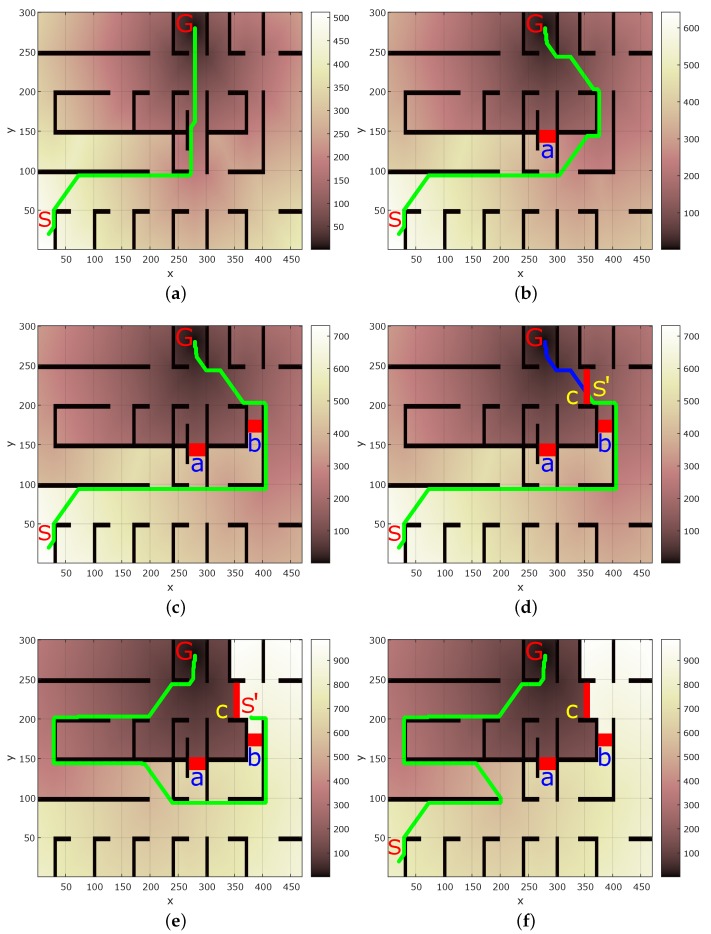
D* planned paths from S to G. (**a**) Path without obstacles; (**b**) Path when knowledge of obstacle a is informed to robot; (**c**) Path when obstacle a and b are informed; (**d**) With a new obstacle c robot stops at $S^{\prime }$. The blue path cannot be navigated; (**e**) New path from $S^{\prime }$ to G; (**f**) With information of obstacle c informed to other robots, a subsequent robot plans a path considering all obstacles to G. The darker shades in the colormap represent proximity to goal (G).

The proposed technique was tested in the simulation environment shown in Figure 4 using the Matlab software and robotics toolkit [43]. D* algorithm [5,6] was chosen for path planning, however, any other algorithm can also be chosen. A grid based navigation is chosen with one unit cost for forward, back, left, and right movement, whereas, for diagonal movement the cost is $\sqrt{2}$ units. In Figure 4, S and G represents the start and goal locations of the robot, respectively.

Figure 4a shows the D* path from start to goal location when there are no obstacles in the map. Figure 4b shows a new obstacle (marked a) in the map found by another robot and communicated to others. The planned path considering this informed obstacle is shown. Similarly, Figure 4c shows the path of the robot which has been informed about both the obstacles marked a and b. For comparison with the traditional method, consider Figure 4d which shows a new obstacle marked c in the map. If no other robot has found this obstacle, the robot starts navigation and stops at location $S^{\prime }$. The path shown in blue color in Figure 4d is not accessible due to the obstacle. The robot then has to plan a new path from the location $S^{\prime }$ to G which is shown in Figure 4e.

It is clear from Figure 4d,e that the robot has to navigate a long distance path: $S\to S^{\prime }\to G$. Moreover, the robot has to plan the path twice at locations S and $S^{\prime }$ which is time consuming. However, if the information about the newly discovered obstacle c is shared with other robots, then subsequently, other robots can consider the obstacle c while planning their paths. As an example Figure 4f shows the path calculated subsequently by a robot considering the informed obstacle (c) from S→G. In traditional robot navigation without the proposed knowledge sharing, the robots would keep wasting time in explicitly discovering obstacles and re-planning paths which is not efficient.

**Figure 5:**
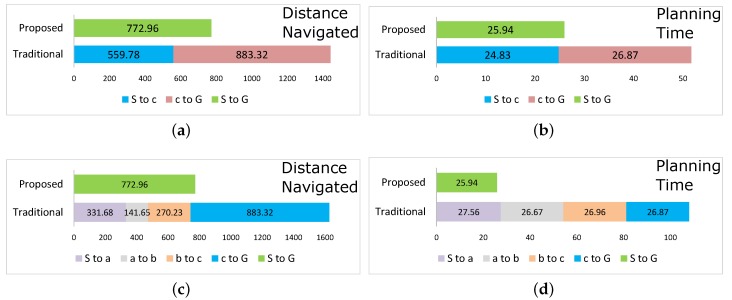
Comparison of proposed vs. traditional method. (**a**,**b**) Distance and time with one obstacle c in Figure 4d,e vs. Figure 4f; (**c**,**d**) Distance and time with three obstacles a–c in Figure 4.

Figure 5a shows the comparison of proposed knowledge sharing and traditional technique for a subsequent robot’s navigation (after the obstacle information has been shared). Figure 5a shows that in traditional navigation, a robot navigates $S\to S^{\prime }\to G$ path, covering a total of 1443.1 units. On the other hand, in the proposed method, the subsequent robot directly navigates S→G path shown in Figure 4f traveling a total distance of 772.96 units. Figure 5b shows the time taken in path planning for the traditional and proposed method for the subsequent robot. Traditional method required planning twice, taking a total of 51.7 s. Whereas, the proposed method required planning only once taking 25.94 s. Thus, the proposed technique takes only 53.56% navigation, and 50.17% planning time of the traditional method.

If we consider the case with all the three new obstacles a–c, a robot would navigate a path S→a→b→c→G. However, the robot would communicate all the obstacle information to other robots. A subsequent robot would then directly take the path S→G shown in Figure 4f. For this case the comparison of total navigation distance and planning time for traditional and proposed method is shown in Figure 5c,d, respectively. The proposed method takes 47.51% of navigation distance, and 24% planning time of the traditional method.

**Figure 6:**
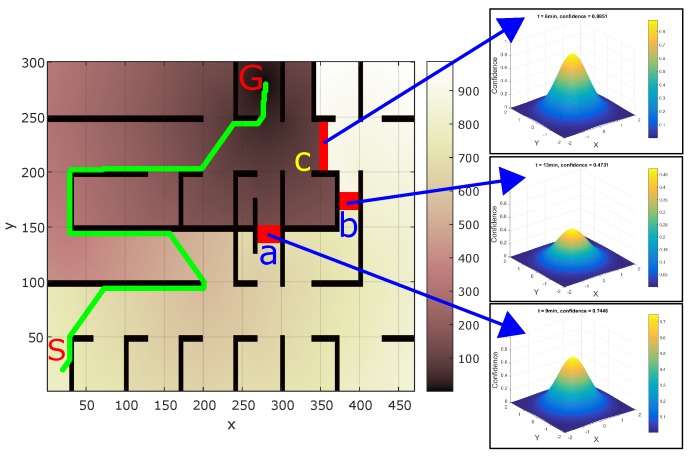
Confidence value (Equation (3)) corresponding to the three obstacles at a particular instance.

Choosing the threshold confidence ($c_{th}$) as 0.55, threshold time ($t_{th}$) as 12 min, and $t_{z}$ as 18 min, the value of *n* in Equation (3) was calculated as 1.9694 from Equation (4). Assuming that obstacle b was discovered first in the map followed by obstacle a and then obstacle c, and assuming that 13, 9, and 6 min, respectively, have elapsed since their update in the map, Figure 6 shows the respective confidence values for the three obstacles given by Gaussian distribution whose peak is equal to the confidence value. The width and breadth of the Gaussian distribution denote the uncertainty in obstacle position. The confidence peaks for obstacles a–c shown in Figure 6 are 0.7446, 0.4731, and 0.8851, respectively. With this configuration, if a robot does not have a time-critical task, it will chose the path through obstacle b as it has the lowest confidence indicating its possible removal. In real world, it may be possible that the obstacle is still present. In that case, the confidence for obstacle b will be reset to maximum value of 1, and robot will explore other paths.

### 4.2. Results in Real Environment

**Figure 7:**
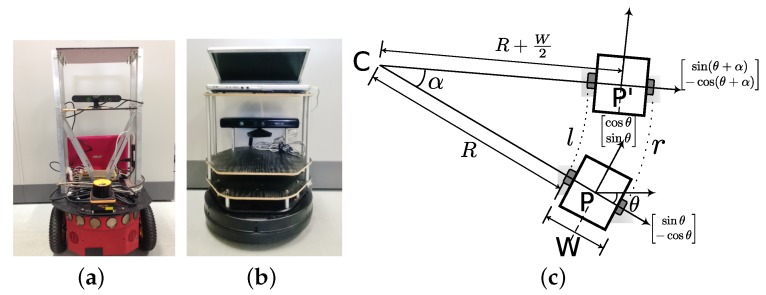
Robots used in the experiments. (**a**) Pioneer P3DX; (**b**) Kobuki Turtlebot; (**c**) Two wheel differential drive motion model.

We performed results in a real environment with Pioneer P3DX [44] and Turtlebot [45] robot shown in Figure 7 using the data-set of [46]. Both the robots were on the same wireless network and could communicate with each other. Grid map of the environment was made using particle filter and modified standard ROS (Robot Operating System [47]) mapping library [48]. Both the robots are two wheeled differential drive robots. We first explain the motion model of the robots. As shown in Figure 7c, *W* is the distance between the two wheels. Let the robot pose at point *P* be given as [x,y,θ]. The angle α as shown in Figure 7c is,

(7) $$\begin{array}{cc} r & =\alpha \;\cdot \;(R+W),\\ l & =\alpha \;\cdot \;R\\ ∴\alpha & =\frac{r-l}{W} \end{array}$$

and the radius of turn *R* as,

(8) $$\begin{array}{c} R=\frac{l}{\alpha },\alpha \neq 0. \end{array}$$

The center of rotation *C* is given as,

(9) $$\left[\begin{array}{c} Cx\\ Cy \end{array}\right]=\left[\begin{array}{c} x\\ y \end{array}\right]-\left(R+\frac{W}{2}\right)\;\cdot \;\left[\begin{array}{c} \sin\theta \\ -\cos\theta \end{array}\right]$$

The new heading $\theta^{\prime }$ is,

(10) $$\theta^{\prime }=\left(\theta +\alpha \right)\;\mathrm{mod}\;2\pi ,$$

from which the coordinates at the new position $P^{\prime }$ are calculated as,

(11) $$\left[\begin{array}{c} x^{\prime }\\ y^{\prime } \end{array}\right]=\left[\begin{array}{c} Cx\\ Cy \end{array}\right]-\left(R+\frac{W}{2}\right)\;\cdot \;\left[\begin{array}{c} \sin\theta^{\prime }\\ -\cos\theta^{\prime } \end{array}\right],\;\;\alpha \neq 0\;\Rightarrow r\neq l.$$

If r=l, i.e., if the robot motion is straight, the state parameters are given as,

(12) $$\theta^{\prime }=\theta ,$$

and,

(13) $$\begin{array}{c} \left[\begin{array}{c} x^{\prime }\\ y^{\prime } \end{array}\right]=\left[\begin{array}{c} x\\ y \end{array}\right]+l\;\cdot \;\left[\begin{array}{c} \cos\;\theta \\ \sin\;\theta \end{array}\right],\;\left(l=r\right). \end{array}$$

**Figure 8:**
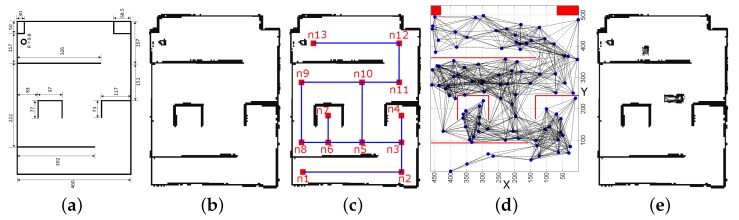
(**a**) Dimensions of the test environment; (**b**) Grip map; (**c**) Node map; (**d**) PRM result; (**e**) Updated grid map with new obstacles.

Figure 8a shows the dimensions of the actual environment used for the experiment. Figure 8b shows the grid map of the environment without the dynamic obstacles. Both the robots had this map before starting the experiment. The node representation of the map is shown in Figure 8c. PRM path planning was used in the experiment and the PRM nodes are shown in Figure 8d.

The threshold confidence $c_{th}$ was set to 0.45, the threshold time $t_{th}$ to 60 s, and $t_{z}$ was set to 150 s. From Equation (4), the value of the degree of the decay curve *n* was calculated to 0.652.

**Figure 9:**
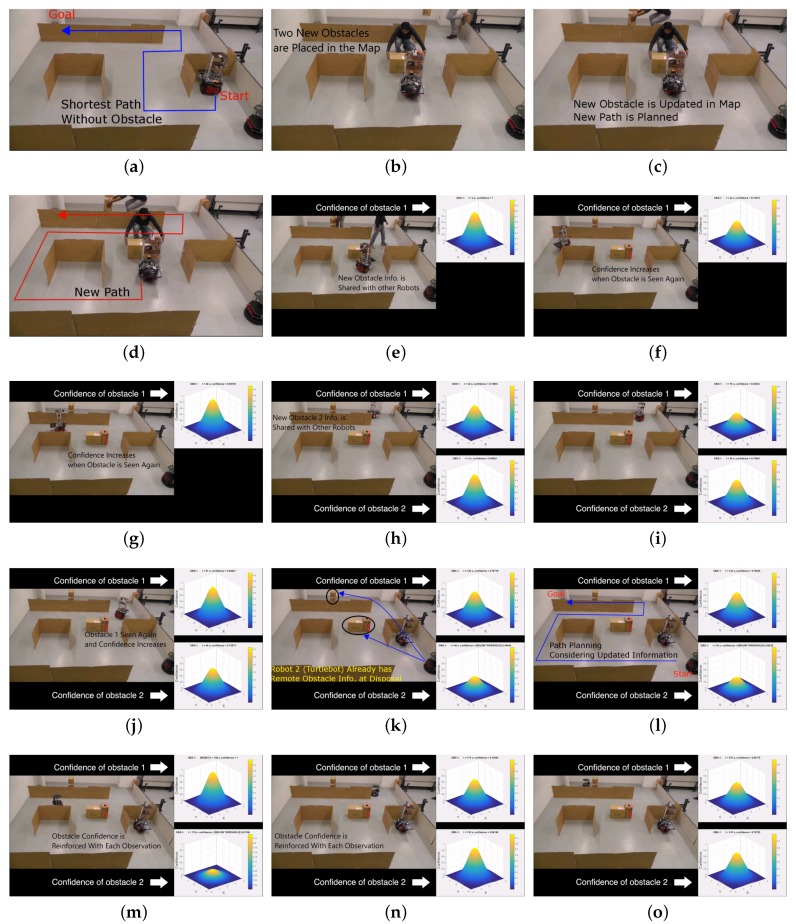
Experiments in real environment. (**a**) Shortest path from start to goal without obstacles; (**b**) Two dynamic obstacles are placed in the map; (**c**) P3DX updates the map with new obstacle; (**d**) New path planned by P3DX; (**e**) P3DX shares the new obstacle information with Turtlebot. Confidence of obstacle is tracked; (**f**) Obstacle 1 confidence decays with time; (**g**) Confidence of obstacle 1 increases when it is seen again; (**h**) Obstacle 2 is observed, updated in map and shared with Turtlebot; (**i**) Obstacle 1 confidence falls down; (**j**) Obstacle 1 is seen again and confidence is restored; (**k**) Turtlebot has information about the two remote obstacles; (**l**) Turtlebot plans a path considering the shared obstacle information; (**m**) Obstacle 1 confidence is reinforced with each observation; (**n**) Obstacle 2 confidence is reinforced with each observation; (**o**) Turtlebot reaches its goal. See Table 1.

Video of the experiment can be downloaded from the link provided in the Supplementary Materials section. Figure 9 shows the various stages of the experiment. In the experiment, the start and goal locations of the robot are indicated in Figure 9a. Both the robots had the grid map of the environment beforehand. Initially, P3DX was programmed to navigate towards the goal and come back to its starting position. Until then, Turtlebot robot was programmed to remain stationary.

The shortest path calculated by P3DX is shown in Figure 9a. Once P3DX starts its navigation, two new obstacles are placed as shown in Figure 9b. P3DX updates the map with new obstacle shown in Figure 9c. It plans a new path towards the goal shown in Figure 9d. P3DX shares the new obstacle information with Turtlebot and tracks the confidence of the obstacle (Figure 9e) which decays with time, shown in Figure 9f. As explained earlier in Section 3, when obstacle 1 is observed again at the same position, its confidence is reset to one. This is shown in Figure 9g in which P3DX resets the confidence threshold to one and this information is also shared with Turtlebot. Figure 9h shows that one more obstacle 2 observed by P3DX and its information is also shared with Turtlebot. Figure 9i,j shown the resetting of obstacle’s confidence values upon observing it again. The map updated by P3DX is shown in Figure 8e.

The dictionary of edge information (E) transferred by P3DX to Turtlebot is (based on the node map of Figure 8c),

(14) $$E=\{\;\cdots \left\{E_{5-10}:1\right\},\left\{E_{12-13}:-1\right\}\cdots \;\}.$$

This minimal information is enough to convey Turtlebot that there are two obstacles between nodes $n_{5}$ and $n_{10}$, and between nodes $n_{12}$ and $n_{13}$ as shown in Figure 8c. Moreover, it is also conveyed that the first obstacle is not traversable (indicated by value of 1), while the other is traversable (indicated by value of −1).

In the proposed knowledge sharing scheme, the Turtlebot already has the information of the two new obstacles with their confidence values beforehand, shown in Figure 9k. Hence, when Turtlebot is instructed to go to the goal location, it plans a path considering the shared information from P3DX robot. The path planned by Turtlebot considering the shared obstacle information is shown in Figure 9l. Notice that the confidence of obstacle 1 is reset when it is observed again (Figure 9m) and Turtlebot shares this information with P3DX. Similarly, obstacle 2 confidence is updated (Figure 9n) when observed again. Turtlebot thus reaches its goal in an efficient manner considering the shared information about obstacles from P3DX (Figure 9o). In the absence of the proposed scheme, Turtlebot would have relied on the old map information and would have to stop at the first obstacle and then re-plan a new path to the goal. This was avoided in the proposed method. Although the experiment environment was small, if we consider a large environment, it is evident that sharing obstacle information would save more time and navigation. This would also turn into battery power efficiency of the robots. The values of obstacle confidence at different times are summarized in Table 1.

## 5. Discussion

From the results in simulation and real experiments, it is evident that sharing obstacle information can bring efficiency in robot navigation in a multi-robot system. In fact, this scheme attempts to mimic the symbiotic behavior of people in large environments. People often share such information with other people at different levels of abstraction, viz. ‘Hey, they blocked the way in front of the library’, enabling other people to use this information to plan an alternate path. This knowledge sharing is particularly useful in large maps. Notice that, in all cases, the first robot to find a new obstacle blocking a path needs to re-plan a new route to the goal in both traditional and the proposed navigation method. However, in the proposed method, the subsequent robots benefit from information shared by other robots for better path planning. Moreover, each robot updates the obstacle information whenever it is observed again and shares the information with other robots.

One significant factor in multi-robot communication is the amount of data exchanged. The proposed node representation of the path enables that minimum communication is sufficient to convey meaningful messages. A lazy update of obstacle information by robots ensures that the robots only stop to update the relevant information which affects the current navigation towards their goals.

The proposed scheme brings several merits in multi-robot path planning and navigation, nevertheless, certain issues remains to be addressed. For example, the proposed method does not transfer the exact dimensions of the obstacle. A blocked path which cannot be traversed by one robot might actually be traversable by another robot of narrow dimensions. The proposed scheme does not take the dimensions of the robots and obstacle geometry into consideration. Although it is possible to also incorporate such information, it has not been done in our experiments to save communication bandwidth and computation.

It is also important to initially set the parameters of the decay curve to model the transience of obstacles. The parameters of the decay curve depends on factors like the size of the service area, number of service robots deployed, and diversification of their service locations. For example, if the service area is small, there is a high probability for one of the robots to observe (or update, or remove) an obstacle. In such case, a small value of threshold time ($t_{th}$) and threshold confidence ($c_{th}$) will suffice. Moreover, if a shortest path is notified to be blocked, a robot with no time-critical task at hand can traverse the shortest path to confirm the obstacle’s existence. For example, in case of Figure 4f, the shortest path is blocked by obstacle ‘a’. However, a robot with less time-critical task can take the shortest path to confirm the existence of the obstacle and accordingly notify other robots. The initial values can be set empirically, and later the appropriate values can be set by statistically analyzing (for ex. clustering) the time of existence of the obstacles at a particular location. In the proposed scheme, an obstacle is assumed to be removed if its confidence falls below a threshold. If it still exists, the first robot to observe it suffers from re-planning the path, however, since the information is shared, the subsequent robots benefit ultimately. The threshold values can also be dynamic, i.e., threshold increased or decreased, depending upon the nature of obstacles at specific times.

## 6. Conclusions

This paper proposed a method in which multiple robots share information about the locally encountered obstacles in the path. This allows robots to get timely information about remote locations in the map without having to explicitly visit those areas. To overcome the problems of correspondence problem in different maps build by the robots, the paths of the map were presented as nodes. The robots only need to share if certain edges are blocked. This allows to convey the information to different robots by consuming minimum communication bandwidth. The transient nature of temporary obstacles was modeled through which the robots can keep a track of which obstacle might have been removed. To this end, a decay function was proposed whose parameters can be calculated for different types of environments. Experiment results confirm that, in the long run in large environments employing multiple robots, the proposed method can improve the efficiency of the system in terms of shorter distance traveled by the robots, and shorter planning time by eliminating path re-planning. The proposed method requires networking between the robots which is the only overhead. However, most of the buildings already have facilities of wireless networks, and robots can use pre-existing infrastructure to their benefit. The proposed method can easily be extended to outdoor robots and automobiles like cars which have navigation systems installed and useful real-time information about different paths can be provided.

## Figures and Tables

**Table 1:** Confidence (*c*) of obstacles at different times in Figure 9. Values in red represents confidence below threshold ($c_{th}=0.45$).

	Obstacle 1		Obstacle 2		
Figure	Time (s)	Confidence	Time (s)	Confidence	Remark
Figure 9e	0	1.00	−	−	Obs.1 observed
Figure 9f	23	0.70	−	−	−
Figure 9g	25	0.96	−	−	Obs.1 confidence reset
Figure 9h	42	0.75	2	0.94	Obs.2 observed
Figure 9i	76	0.50	35	0.75	−
Figure 9j	81	0.94	40	0.72	Obs.1 confidence reset
Figure 9k	120	0.78	80	0.44	−
Figure 9l	123	0.75	83	0.42	−
Figure 9m	158	1.00	118	0.23	Obs.1 confidence reset
Figure 9n	175	0.75	135	0.96	Obs.2 confidence reset
Figure 9o	187	0.65	147	0.79	−

## Supplementary Materials

Video of the experiment can be downloaded from: http://www.mdpi.com/1424-8220/17/7/1581/s1.

## Abbreviations

The following abbreviations are used in this manuscript:

SLAM	Simultaneous Localization and Mapping
PRM	Probabilistic Roadmap Planner
